# Emerging Nanochitosan for Sustainable Agriculture

**DOI:** 10.3390/ijms252212261

**Published:** 2024-11-15

**Authors:** Xia Wang, Maolin He, Xueli Wang, Song Liu, Lin Luo, Qin Zeng, Yangjin Wu, Yinan Zeng, Zhonglin Yang, Guoqiang Sheng, Ping Ren, Han Ouyang, Rong Jia

**Affiliations:** 1 The Key Laboratory of Land Resources Evaluation and Monitoring in Southwest China, College of Geography and Resources, Sichuan Normal University, Chengdu 610066, China; wangxia114@mails.ucas.edu.cn (X.W.); yangzhonglin@stu.sicnu.edu.cn (Z.Y.); renping@sicnu.edu.cn (P.R.); 2 School of Nanoscience and Engineering, University of Chinese Academy of Sciences, Beijing 100049, China

**Keywords:** nanochitosan, sustainable agriculture, renewable resources, antimicrobial, delivery system, SDGs

## Abstract

Chemical-intensive agriculture challenges environmental sustainability and biodiversity and must be changed. Minimizing the use of agrochemicals based on renewable resources can reduce or eliminate ecosystems and biodiversity threats. Nanochitosan as a sustainable alternative offers promising solutions for sustainable agricultural practices that work at multiple spatial and temporal scales throughout the plant growth cycle. This review focuses on the potential of nanochitosan in sustainable agricultural production and provides insights into the mechanisms of action and application options of nanochitosan throughout the plant growth cycle. We emphasize the role of nanochitosan in increasing crop yields, mitigating plant diseases, and reducing agrochemical accumulation. The paper discusses the sources of nanochitosan and its plant growth promotion, antimicrobial properties, and delivery capacity. Furthermore, we outline the challenges and prospects of research trends of nanochitosan in sustainable agricultural production practices and highlight the potential of nanochitosan as a sustainable alternative to traditional agrochemicals.

## 1. Introduction

Agriculture represents one of the cornerstones for human development. However, current agriculture often relies on the massive use of fertilizers and pesticides that adversely affect living organisms and ecosystems [1]. It is an important sustainable development goal (SDG) to achieve the environmentally sound management of chemicals and all wastes and significantly reduce their release into air, water, and soil in order to minimize their adverse impacts on human health and the environment [2]. Sustainable agricultural production patterns are a new trend of future agricultural development. Sustainable agriculture aims to provide sufficient nutritious food for all, while reducing environmental and health risks simultaneously [3]. The minimization of agrochemicals represents a fundamental tenet of sustainable agriculture, with the objective of reducing or eliminating adverse effects on living organisms and ecosystems [4]. Sustainable agriculture relies on the support of advanced technologies to provide more sustainable pesticides, fertilizers, and materials [5].

Nanomaterials and nanotechnologies offer new opportunities to address the challenges associated with agroecology [6]. Nanomaterials have a small size, tunable surface chemistry, and high-efficiency properties as a key driver in accelerating future sustainable agriculture [6]. Nanomaterials have been used to develop adsorbents, fertilizer agents, catalysts, antibacterial substances, and delivery systems. Among the available natural substances, nanochitosan is a biocompatible and biodegradable polymer with enormous structural modification potential that is expected to promote the development of sustainable agriculture [7].

Nanochitosan is derived from chitin, a critical biodegradable biomass polysaccharide, and is obtained by the chemical deacetylation of chitin under alkaline conditions. It can be obtained from renewable resources [8]. Chitin is the largest untapped renewable resource and one of the richest polymer polysaccharides in nature [5,9]. Nanochitosan exhibits multiscale architectures based on random copolymers of glucosamine and N-acetylglucosamine units and demonstrates a diverse range of morphologies. These include small oligomers, rod-shaped nanocrystals, elongated nanofibers, and hierarchical assemblies of nanofibers [10]. Nanochitosan is a non-toxic, bioadhesive, and biocompatible compound that is bioavailable and biodegradable [11,12]. The United States Food and Drug Administration has designated nanochitosan as a “generally recognized as safe” food additive [13]. A variety of distinct nanochitosan derivatives have been synthesized and applied in a diverse range of fields, including agriculture, food, textiles, and medicine [10,14]. Nanochitosan is a versatile and biocompatible material with the potential to be utilized throughout the entire crop growth cycle. It can be employed as a soil conditioner, plant growth regulator, vegetable and crop disease management agent, fruit antistaling agent, and seed coating agent. Nanochitosan is easily degraded in the environment, preventing the accumulation and enhancing the quantity of organic matter, thereby improving soil structure and health due to its high biodegradability [15,16]. In addition, nanochitosan can absorb and retain nutrients in the soil, including nitrogen and phosphorus, which are particularly beneficial when highly absorbent. Finally, nanochitosan is also anticipated to regulate plant pathogens and enhance crop protection through antimicrobial and biopesticide properties [17,18]. Nanochitosan can regulate the release and site-specific delivery of active ingredients by reducing the toxicity of pesticides to plants, enhancing their absorption, and improving solubility and stability [19]. The controlled release of its active ingredients can mitigate environmental threats to ecosystems and human health by enhancing the bioavailability of pesticides. These properties render nanochitosan an optimal material for the promotion of agroecology [7]. The mechanism of chitosan nanoparticles enhancing crop yield and mitigating plant diseases mainly involves the following aspects: firstly, as a soil conditioner, it regulates the soil microbial structure and improves soil quality; secondly, it directly regulates crops to promote growth; and finally, it directly kills pests and diseases or acts as a drug carrier.

**Figure 1:**
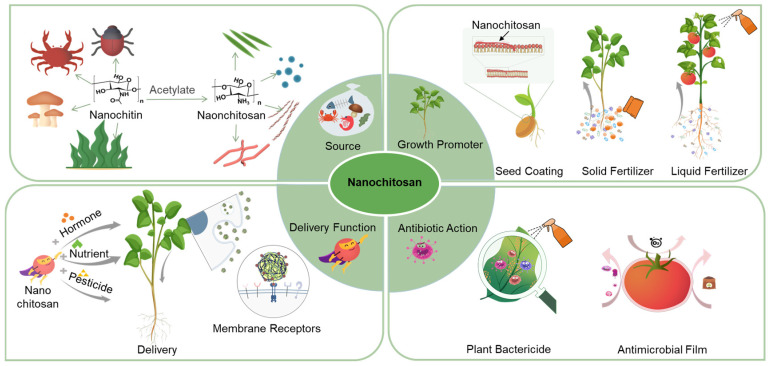
Summary of the nanochitosan origin and application in multiple spatial and temporal scales throughout the plant growth cycle.

This review discusses the sources, applications, mechanisms, and prospects of nanochitosan in agroecology and clarify the necessity for the advancement of agroecology (Figure 1). We also summarize the mechanism of action and application scenarios of nanochitosan throughout the entire crop cycle, including its effects on crop yield, pesticide activity, and the reduction in agrochemical accumulation. Furthermore, the sources of nanochitosan and its effects on plant growth, antimicrobial properties, and delivery capacity are classified and discussed in detail. Finally, we discuss the challenges and provide an outlook for future nanochitosan research in agroecology, as well as the focus and trends.

## 2. Source and Preparation of Nanochitosan

### 2.1. Source of Chitosan

Nanochitosan is derived from the chemical deacetylation of chitin under alkaline conditions. Chitin, a polymer found throughout the biosphere, is the second most abundant polymer after cellulose [20]. In more than 90% of animal species and insects, the primary component of the arthropod exoskeleton is a chitin-based complex. Currently, the majority of chitin production is derived from crab and shrimp shells from the canning industry, which avoids environmental pollution and nutrient waste [21]. Chitin is currently produced on a global scale, with an estimated annual production of between 10^10^ and 10^12^ tons [22].

### 2.2. Morphological Classification and Preparation Method of Nanochitosan

Nanochitosan encompasses chitosan nanofibers, nanoparticles, and nanocrystals that can be prepared by the deacetylation of chitin [23]. Chemical, enzymatic, microwave, and microbiological methods have been employed to prepare nanochitosan [24,25]. The chemical methods and processes involved are more straightforward and quicker, and the products have been shown to have more potent bioactivities. Furthermore, nanochitosan can be extracted through a combination of microbiological and biotechnological methods, including those involving *Mucor rouxii*, *Absidia butleri*, *Abesidiacorrulea*, *pyrioceana blue*, and waste citric acid bacteria [26,27]. The microbiological and biotechnological methods have considerable potential for development in the field of agroecology, due to its low energy and resource consumption, eco-friendliness, and high efficiency.

## 3. Nanochitosan Promotes Plant Growth

Nanochitosan is an environmentally friendly alternative to synthetic chemical agents for plant growth promotion due to its biocompatibility and biodegradability. This section aims to provide an overview of the progress of research on nanochitosan as a plant growth promoter (Table 1).

### 3.1. The Mechanisms of Nanochitosan Enhancing Plant Growth

The chitosan polymer exhibits a high density of positive charges and a profound affinity for cell membranes due to the presence of one amino group (-NH_2_) and two hydroxyl groups (-OH) within its monomeric structure [30]. The amino group confers a net positive charge to chitosan, enabling it to effectively bind with macromolecules, negatively charged lipids, and proteins. Chitosan can also form coordination bonds with metal ions to produce complexation and bind together. The hydroxyl functional group also serves as an electron acceptor, facilitating metabolic processes and signal transduction through binding to cellular receptors.

Nanochitosan has been demonstrated to enhance plant resistance to abiotic stress by inducing the production of key defense enzymes, including catalase, polyphenol oxidase, phenylalanine ammonia-lyase, and peroxidase. These enzymes are crucial in protecting plant cells from oxidative damage caused by reactive oxygen species [31]. Phenylalanine deaminase can induce plant metabolism. Polyphenol oxidase can catalyze phenolic substances to synthesize lignin, the raw material for building cell wall structure. In addition, nanochitosan can reduce chloroplast membrane disruption and increase chlorophyll content and the total photosynthesis of plants. This is achieved by reducing ethylene concentration and inhibiting chlorophyll enzyme activity [32]. Following the application of chitosan to leaves, α-amylase activity, chlorophyll content, and endogenous hormone content can be adjusted to further alter stress resistance and transpiration, thereby effectively increasing plant biomass. The expression of nitric oxide, an important signaling molecule in plant defense mechanisms, was significantly increased following exposure to nanochitosan [33].

Nanochitosan naturally serves as a source of carbon, oxygen, nitrogen, and phosphorus nutrients for plants. Moreover, nanochitosan as a nanocarrier can also support plant macronutrients (calcium, potassium, nitrogen, sulfur, phosphorus, and magnesium) and micronutrients (manganese, copper, nickel, zinc, boron, iron, and chloride) through its functional groups [34]. The cationic properties of chitosan can be employed to enhance the anion exchange capacity of the soil or growing medium, which is usually much lower than the cation exchange capacity. The leaching of anionic nutrient fertilizers (e.g., nitrates and phosphates) in soils treated with chitosan can be reduced [35]. 

### 3.2. The Application of Nanochitosan on the Plant Growth Cycle

**Figure 2:**
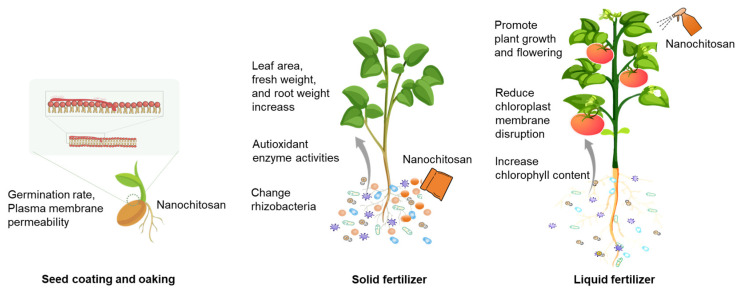
The application of nanochitosan during the plant growth cycle.

Nanochitosan as a sustainable alternative offers promising solutions for sustainable agricultural practice that work at multiple spatial and temporal scales throughout the plant growth cycle (Figure 2).

Nanochitosan is an appropriate seed coating material due to its favorable film formation, adhesion, and hygroscopicity properties. Nanochitosan can form a seed coating on the seed surface, alter the permeability of the seed plasma membrane, and accelerate the germination and germination rate. The germination rate, mean germination time, germination value, picking value, and mean daily germination rate of seeds were affected by the nanochitosan environment. For example, chickpea seeds treated with chitosan from thiamine nanoparticles exhibited 90% germination compared to 75% germination with water. The nanochitosan treatment resulted in a reduction in the average germination time of pepper seeds by 4.9 to 5.3 days. The treatment promoted seedling emergence and growth as well as improved pepper growth parameters, including total leaf area, fresh weight, and root weight [36].

The application of a nanochitosan coating resulted in enhanced seed tolerance to abiotic stress, as evidenced by increased peroxidase, catalase, phenylalanine ammonia-lyase, and malondialdehyde (MDA) levels, which serve as indicators of lipid peroxidation. The MDA was also reduced by the increased antioxidant activity. Seedlings coated with nanochitosan resulted in elevated levels of defense enzymes and a tenfold increase in auxin content [37]. Chitosan-soaked groundnut seeds showed significant increases in germination, lipase activity, gibberellin, and indoleacetic acid levels [38]. Nanochitosan–glycinebetaine pretreatment reduced the relative permeability of the maize plasma membrane under heat and drought stresses conditions, reduced the damage of heat stress on maize, and also to improved drought hardiness [39]. It is worth noting that the optimal concentration of nanochitosan was found to be 30 ppm, while nanochitosan at a concentration of 90 ppm can lead to adverse effects on the majority of the traits studied [40]. 

Nanochitosan can also be sprayed on the leaf surface to promote plant growth and flowering due to its better dispersibility and cell affinity. Nanochitosan is often classified as an elicitor for activating plant genes underlying secondary metabolite biosynthesis pathways. Nanochitosan was found to increase plant resistance to *S. litura* by activating endogenous signaling cascades (Ca^2+^ influx and phytohormone accumulation) and enhancing the production of defense metabolites (such as sesamolin and shanzhiside methylester) via upregulating defense metabolite biosynthetic genes. The highest resistance of sesame plants to *S. litura* and the alleviation of plant oxidative stress by increasing the activities of antioxidant enzymes were achieved by the foliar application of 100 mg L^−1^ nanochitosan [41].

Nanochitosan has also been demonstrated to be an effective agent for combating the common plant pathogen *Xanthomonas*. It can be sprayed on plants to induce the accumulation of bioactive secondary metabolites. Foliar applications of chitosan during flowering can increase the number of flowers, the weight per plant, and the yield per forage of chamomile. Low concentrations of chitosan (10 mg L^−1^) were found to effectively induce vegetative development in *Brassica napus*. The foliar application of chitosan has been demonstrated to promote tomato growth and affects the expression of endogenous chitinase-encoding genes and mycorrhiza formation [42]. The application of lactic acid chitosan to the foliage of plants has been demonstrated to promote the accumulation of bioactive substances [43]. Nanochitosan at a concentration of 2–4 g L^−1^ promotes the growth of maize plants by increasing the chlorophyll content, α-amylase activity, and endogenous hormone content of maize seedling leaves [44]. The rise in organic acids indicated an enhanced stress resistance mechanism in maize plants following an enhanced nanochitosan treatment. The foliar spray application of nanochitosan is a viable method for cultivating medicinal and aromatic plants, which are known to possess superior health benefits and medicinal properties. This practice effectively stimulates the accumulation of selected phenolic compounds in basil and lemon balm [43].

In addition to seed coating and foliar spraying, nanochitosan can also be added into the soil. The application of nanochitosan at a rate of 6 mg kg^−1^ in soil has been demonstrated to enhance nitrogen accumulation, dry matter, grain yield, crude protein concentration, and the translocation of nutrients from vegetative organs to grains following anthesis. The positive effect of nanochitosan is attributed to the activation of PEPC (phosphoenolpyruvate carboxylase), SPS (sucrose phosphate synthase), and crucial metabolic enzymes in flag leaves and spikes [45]. It can be used in a sustainable biofertilization strategy that enhances plant growth and improves health-promoting compounds in wheat. The combination of nanochitosan with *Bacillus* and *Rhizobium* has been demonstrated to have a growth-promoting effect on maize. After treatment with nanochitosan, seed germination significantly increases from 60% to 96.97%. Additionally, the plant height, leaf area, and the alcohols and acids in plant metabolites exhibited notable enhancements [29]. Notably, the content of esters and aldehydes exhibited a considerable increase. The application of nanochitosan (40 mg L^−1^) in conjunction with Pseudomonas spp. resulted in a notable enhancement of plant vigor, including an increase in plant height, chlorophyll, and carotenoid content in maize plants. Soil enzyme activities, including dehydrogenase and alkaline phosphatase, were significantly increased. The combination of nanochitosan with Bacillus spp. in maize has been demonstrated to enhance plant height, leaf area, seed germination, and organic acid production in response to stress tolerance [29]. The application of nanochitosan has been demonstrated to contribute to the bioremediation of heavy-metal-contaminated soils. Nanochitosan can improve the Cd phytoremediation capacity of *Datura stramonium* L. The link of nanochitosan with κ-carrageenan has been shown to effectively remove and immobilize Cd^2+^ from both water (750.2 μmol/g) and soil (992.7 μmol/g) [46,47]. 

## 4. The Antimicrobial Properties of Nanochitosan

Nanochitosan exhibits excellent antimicrobial properties and significantly inhibits various bacteria, fungi, and viruses [5]. Since Allen [48] first proposed the broad-spectrum antibacterial activity of chitosan, the antibacterial properties of chitosan and its derivatives have been a subject of great interest to researchers. This section aims to provide an overview of the research progress of nanochitosan as an antimicrobial agent and its potential applications in agriculture. 

### 4.1. The Antibacterial Mechanisms of Nanochitosan 

**Figure 3:**
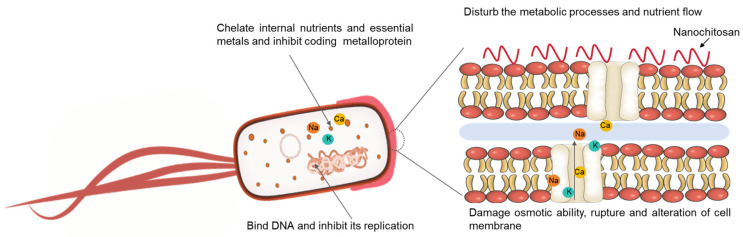
The antibacterial mechanisms of nanochitosan.

Nanochitosan exhibits a variety of inhibitory effects against bacteria, fungi, and viruses. A nanochitosan polymer has one amino group (-NH_2_) at the C-2 location and two hydroxyl groups (-OH) at the C-3 and C-6 locations. The -NH_2_ group imparts a net-positive charge to nanochitosan, allowing it to interact with anionic molecules, including structural and functional anionic protein and cellular membrane phospholipids. It is notable that the C6-OH group has hyperactive -OH groups that can rotate freely with minimal steric hindrance. The -OH functional group acts as an electron acceptor and hastens signal transduction by engaging with cell receptors and accelerating metabolic events. Consequently, nanochitosan has four primary modes of action: (1) The metabolic processes and nutrient flow of the microorganism are disrupted by the formation of a dense polymeric membrane on the pathogen’s surface, resulting from the deposition of chitosan [49]. (2) It interacts electrostatically with the cell membrane, causing increased permeability of the pathogenic organism’s cell envelope, rupture and alteration of the cell membrane, osmotic damage, and eventual death [50]. (3) It forms complexes with certain metal ions, inhibiting the metalloproteins’ activity and microbial growth [51]. (4) It binds to negatively charged phosphate groups in the nucleic acid backbone, thereby inhibiting mRNA transcription and protein translation [52]. This results in the release of LPS from the outer membrane, thereby increasing the permeability of the outer membrane (Figure 3). 

In addition to nanochitosan’s direct effect on pathogenic bacteria, nanochitosan also has an indirect effect on enhancing plant resistance to pathogenic bacteria. Nanochitosan has been demonstrated to effectively induce a host defense response against plant pathogens and generate systemic resistance [53]. Nanochitosan can induce plant defense mechanisms against a variety of plant pathogens, including plant viruses [54]. It can inhibit phage-induced infection through inactivation at the cellular level and the inhibition of propagation [55]. Nanochitosan has also been shown to upregulate the plant gene functions associated with pathogenicity-related genes, such as PR proteins (PR-1 and PR-2 (β-1,3-glucanase), PR-8 (chitinase), and PR-10) and antioxidant genes. The activation of defense genes leads to the accumulation of various enzymes and stress-specific metabolites. (1) In one study, nanochitosan increased the levels and activities of antifungal (PR-1) and hydrolytic enzymes (β-1,3-glucanases (PR-2) and chitinases (PR-8)) to degrade microbial cell walls [56]. (2) Nanochitosan increased the polyphenol oxidase content, which catalyzes the synthesis of lignin from phenolics. This process contributes to the formation of cell wall structures that serve as a barrier to pathogen penetration [57]. (3) Nanochitosan increased ribosome-inactivating protein (PR-10), which releases adenine residues from both ribosomal and non-ribosomal substrates, thereby inhibiting the pathogenic translation [56]. (4) Nanochitosan initiates complex signal transduction pathways involving reactive oxygen species (ROS) production, such as peroxidase, catalase, and superoxide dismutase in the host plant [57]. 

**Figure 4:**
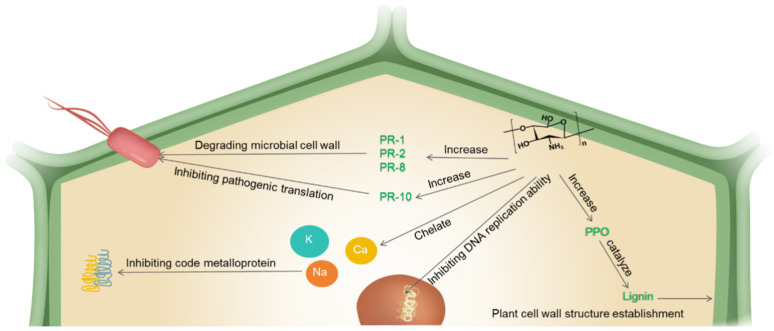
The mechanism of resistance of nanochitosan to plant pathogens.

Notably, the immunostimulatory activity of nanochitosan may not be mediated by a specific receptor-like molecule but rather by the interaction of its surface cations with negatively charged phospholipids. Furthermore, the pathogen is unlikely to develop resistance to nanochitosan, because the negative charge of microbial cell envelopes is evolutionarily conserved and unlikely to be altered by a single gene mutation. Further evidence is needed to clarify the direct activity of nanochitosan against plant viruses. It is reasonable to assume that the antiviral activity of nanochitosan is dependent on its ability to induce plant immune responses [58]. In summary, nanochitosan can induce the host plant to produce defense-related proteins, enzymes, and secondary metabolites that directly or indirectly degrade pathogens. The mechanism of resistance of nanochitosan to plant pathogens is shown in Figure 4.

### 4.2. The Application of Nanochitosan as an Antimicrobial Agent

Nanochitosan has been demonstrated to have inhibitory effects on bacteria and fungi, making it a promising natural antibacterial agent. Nanochitosan’s broad-spectrum antibacterial properties, good biocompatibility, and natural origin make it an attractive candidate for use as an antimicrobial agent in agriculture. Nanochitosan is used to prevent and mitigate bacterial wilt caused by Ralstonia solanacearum. The prophylactic application of nanochitosan solutions has been demonstrated to effectively reduce the incidence and intensity of bacterial wilt disease among potato and tomato plants. The application of a 200 μg mL^−1^ nanochitosan solution resulted in a significant increase in the health rate and health grade of potato plants, from 15.38% and 20.87% to 78.93% and 71.85%, respectively. Furthermore, therapeutic spraying demonstrated a degree of efficacy, although it was less pronounced than that observed with preventive spraying [59]. 

Leaf blight and leaf spot are two bacterial diseases that severely affect rice. Nanochitosan solutions have been demonstrated to exhibit robust antibacterial activity against both of these rice pathogens. Specifically, the application of this type of solution results in a significant reduction in both the incidence and severity of these diseases in rice. Moreover, the activities of phenylalanine ammonia-lyase, peroxidase, and polyphenol oxidase in rice seedlings are significantly increased in the chitosan solution group [60]. The direct antibacterial activity of nanochitosan and its ability to indirectly induce resistance are the primary mechanisms underlying its protective effects against bacterial pathogens in rice. Moreover, the growth of *Acidovorax citrulli*, the bacterium responsible for bacterial fruit blotch in watermelon, was significantly inhibited. Nanochitosan has also been demonstrated to be an effective agent for combating the common plant pathogen *Xanthomonas*. A chitosan solution with a concentration of 100 μg mL^−1^ has been demonstrated to exhibit inhibitory effects on *Xanthomonas* pathogens from diverse origins. The antibacterial activity of chitosan solutions was enhanced by the addition of sodium chloride, irrespective of the nutrient type or sterilization method. Moreover, the chitosan solution demonstrated robust antibacterial activity against the specific strain R22580 of *Xanthomonas axonopodis pv. poinsettiicola* within a pH range of 5.5–7.0. This indicates that chitosan has the potential to be utilized as an antimicrobial agent to independently inhibit plant-pathogenic *Xanthomonas* [61]. 

**Figure 5:**
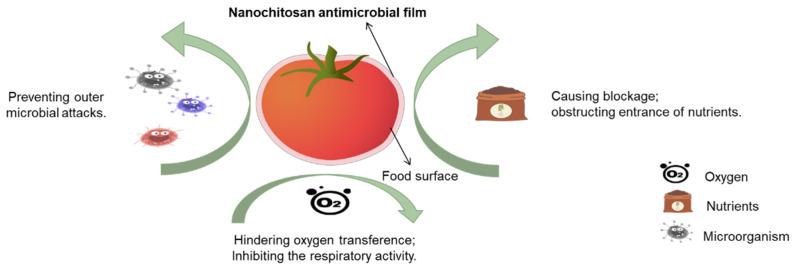
The mechanism of antimicrobial nanochitosan films.

Postharvest decay of agricultural products during transportation and storage causes huge economic losses. Considering the problem of pesticide residues, safer alternatives are needed to control postharvest decay. Nanochitosan has gained considerable acceptance as a dietary supplement and has been approved by the FDA [13]. Nanochitosan is employed as a coating additive for fruits, seeds, and vegetables in edible antibacterial films (Figure 5). The antibacterial bioresorbable materials, composed of poly (lactic acid) and chitosan (4% concentration), have been demonstrated to achieve a sterilization rate of 99% when applied to the BTS [62]. A variety of techniques, including direct casting, coating, extrusion, layer-by-layer assembly, and dipping, can be employed to fabricate nanochitosan-based edible films [63]. It is anticipated that nanochitosan will be utilized in the prevention of postharvest diseases affecting various fruits and vegetable, including cucumbers [64], mushrooms [65], fish, tomatoes, mangos, bananas, and pomegranates [66]. Films based on nanochitosan can be further incorporated with functional substances such as antioxidants, plant extracts, essential oils, and other additives to enhance barrier properties and functionality. Nanochitosan-based edible nanomodifiers are used for functionalizing starch/guar gum biocomposites with superior packaging properties, targeting stringent edible food packaging on fresh cuts. The storage quality in terms of microbial growth, pH change, color attributes, and weight loss is better preserved when it is used as an edible coating on cut apple fruits [67].

Nanochitosan is frequently combined with other functional materials, including biopolymer films, protein-based films, polysaccharide-based films, inorganic material films, synthetic polymer films, extract-based films, and nanochitosan-derivative-based films. The combined utilization of nanochitosan nanoparticles and Zataria multiflora essential oil (ZEO) has been demonstrated to effectively prolong the shelf life of cucumbers. This effect is presumed to be due to the release of bioactive compounds from the nanochitosan. Moreover, the application of a ZEO–nanochitosan coating has been found to enhance the hardness, respiration rate, and DPPH radical scavenging activity of cucumbers, while simultaneously reducing their power. These findings indicate that ZEO–nanochitosan enhances the antioxidant activity of fruits and inhibits microbial growth during storage [64]. One of the most commonly used edible packaging materials is pectin–chitosan film. The effect of the ratio of pectin to nanochitosan on the properties of the film has also been explored. The ratio of pectin to nanochitosan exerts a notable influence on the thickness, mechanical characteristics, water vapor transmission rate, solubility in water, and oxygen permeability of the material. Our findings indicate that when pectin and nanochitosan are combined in a 1:1 ratio, the tensile strength is as high as 8.96 MPa, the solubility in water diminishes to 37.5%, the oxygen permeability is 47.67 cc·mm/m^2^·day, and the water vapor transmission rate diminishes to 0.2052 g·mm/m^2^·day·kPa. Moreover, the pectin–chitosan film displays hydrophobic characteristics and exhibits inhibitory effects on the growth of *Coccidioides*, *Escherichia coli*, *Aspergillus niger*, and *Saccharomyces cerevisiae* [68]. Due to its antimicrobial activity and ability to elicit defense responses, nanochitosan has emerged as a promising treatment option following harvest.

## 5. The Delivery Properties of Nanochitosan

Nanochitosan has many advantages not only in terms of its source, plant growth promotion, and antimicrobial properties but also in terms of delivery of agrochemicals. The nanodelivery systems can provide the targeted and controlled release of agrochemicals and improve the efficiency and intelligence of agrochemical technologies. A variety of chemicals can be encapsulated in nanochitosan, including plant growth regulators, soil nutrients, and pesticides. The delivery systems based on nanochitosan can avoid the overuse of agrochemicals by controlling the release of active ingredients. These delivery systems may also alleviate the challenge of over-pesticide use [69]. 

### 5.1. The Delivery Mechanism of Nanochitosan

**Figure 6:**
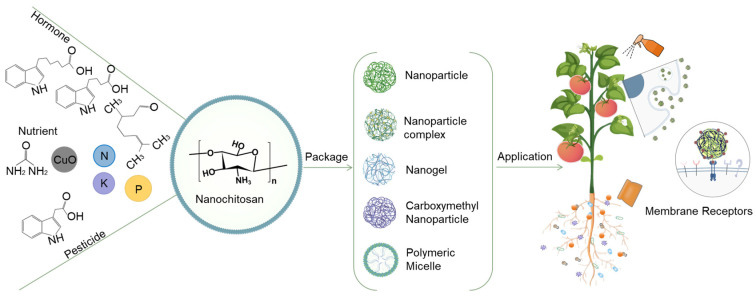
The delivery of the nanochitosan and its derivatives.

Nanochitosan can function well at the molecular and cellular levels due to its small size, tunable surface chemistry, and high efficiency. Nanochitosan also has analogous structural characteristics to glucosamine, giving it significant advantages as a drug carrier. The accessibility of functional groups in nanochitosan facilitates the formation of nanochitosan with other polymers and metal ions. Nanochitosan exhibits excellent adsorption, film-forming, permeability, fiber-forming, moisture-absorption, and moisture-retention properties (Figure 6). The active ingredients can be encapsulated or embedded in the matrix of nanochitosan polymers by ionic or covalent intermolecular/internal bonds to form an effective nanodelivery system formulation [70].

Nanochitosan, as a sustained-release agent, can control the release of the active ingredient, maintain the concentration within the effective concentration range, and prolong the duration of effective action. Agrochemicals encapsulated in nanochitosan matrices can be triggered by a multitude of biotic and abiotic stress factors, including insect pests, plant pathogens, weeds, pH, drought, flooding, salinity, and temperature, among others. Environmental factors or enzymatic reactions may lead to the degradation or rupture of the capsule matrix. Specifically, these stresses affect the regulation of agrochemical release by modulating pore diffusion, capsule swelling, degradation, and surface desorption. The controlled release of agrochemicals based on stimulus response in nanoformulations allows for the effective and efficient delivery of these chemicals to the target site [71]. Nanochitosan enhances the circulation of agrochemicals and increases their retention time in plant tissue, resulting in prolonged half-lives (t1/2). This control of the release of active ingredients increases their bioavailability [15]. Nevertheless, nanochitosan remains a promising candidate for large-scale agricultural applications, as there are still some challenges to overcome regarding its use as a drug delivery vehicle, such as low solubility, slow drug release, and poor targeting stability.

### 5.2. The Application of Nanochitosan for Delivery 

Nanochitosan and its derivatives are frequently employed in the field of delivery due to their advantageous properties, including biodegradability, biocompatibility, antibacterial properties, and adhesion. Nanochitosan has been demonstrated to be an effective delivery system for hormones, trace elements, and pesticides, and it has been shown to effectively inhibit diseases and promote growth.

Nanochitosan is employed as a hormone delivery carrier to stimulate plant growth. Anderson Espirito Santo Pereira et al. present an alginate–chitosan and chitosan–tripolyphosphate nanoparticle system that effectively encapsulates the plant growth regulator gibberellic acid. This nanoformulation increases leaf area, chlorophyll, and carotenoid levels, demonstrating improved biological activity compared to the free hormone [72]. Serdar Korpayev et al. reported a non-toxic, organic, solvent-free, and stable nanocarrier system for auxin consisting of chitosan and silver nanoparticles. Nanochitosan loaded with the auxins indole-3-acetic acid (IAA) and indole-3-butyric acid (IBA) was successfully synthesized. This approach is more effective and promising than using free IAA or IBA in conjunction with IBA–nanochitosan or IAA–nanochitosan [73].

Deshpande et al. evaluated the suitability of zinc-complexed nanochitosan as a potential “nanocarrier” for foliar fertilization. Zn–nanochitosan was synthesized using tripolyphosphoric acid as a cross-linking agent. In their study, plants were cultivated in a zinc-deficient sand medium. Upon application of Zn-nanochitosan to the leaves of the plants following flowering, a significant increase in zinc content was observed in durum wheat varieties, with an increase of 27% and 42% observed after five weeks [74]. Nanofertilizers are produced by encapsulating nitrogen (N), phosphorus (P), and potassium (K) within nanochitosan. These nanofertilizers have been demonstrated to exhibit remarkable efficacy in enhancing the nutrient absorption, photosynthesis, and overall growth of coffee plants. In comparison to the untreated control plots, the application of these nanofertilizers resulted in a significant increase in nitrogen content by 17.04%, phosphorus content by 16.31%, and potassium content by 67.50% in the treated plots. Moreover, there was a discernible enhancement in the number of leaves, plant height, and leaf area of the coffee seedlings [75]. Kondal et al. reported the effects of biodegradable nanopolymer urea formulations on soil enzyme activity and microbial communities involved in the potato nitrogen cycle. In comparison to the conventional urea treatment, the nanochitosan–urea composite treatment demonstrated a notable increase in the soil’s available potassium, organic carbon content, and dehydrogenase activity. Biodegradable polymer–urea composites exert a significant influence on the microbiota associated with soil nitrogen dynamics [3].

Nanochitosan with copper oxide on three isolates of *Fusarium oxysporum* infecting cultivated tomato plants was evaluated. The results of the study demonstrated that nanochitosan with copper oxide is capable of controlling fungal spores. Furthermore, the tissue morphology of the treated plants demonstrated no evidence of phytotoxicity [76]. Samar S. Ibrahim et al. employed an ion gel approach to develop a nanocapsule delivery system integrating nanochitosan and nanocellulose. The objective of this system was to precisely control the release of citronella essential oil (CEO) to mitigate the cotton leafworm *Spodoptera frugiperda*. Insecticidal activity tests indicated that all nanoformulations interfered with the normal development of *S. littoralis*. The combination of CEO–nanochitosan exhibited encouraging results, significantly extending the larval and pupal stages compared to the untreated control. Furthermore, notable decreases in pupal weight, adult lifespan, and female fecundity were observed, particularly after exposure to CEO–nanochitosan treatments [77]. 

## 6. The Challenge of Nanochitosan in Eco-Agriculture

Despite the promising results observed in preliminary nanochitosan studies, the combination of different forms of nanochitosan and their derivatives for the precise protection of the entire plant life cycle, from seed to fruit, for different problems is still a major challenge. 

In terms of environmental concerns, there are big research gaps and limitations in nanochitosan applications. Although a large number of articles mention the environmental advantages of nanochitosan, there is a lack of longevity studies and evaluation of the environmental impacts of its long-term, large-scale application, especially its degradation properties and potential ecological risks when it is present in the soil for a long period of time. These are the key points to be studied in the future. The mechanisms by which nanochitosan exerts its effects on plants remain largely unknown, limiting the full range of applications of nanochitosan in plants. It is crucial to reveal and optimize the specific effects of nanochitosan in agrochemical delivery to plants, utilizing advanced molecular biology tools such as transcriptome and proteome profiling. 

In terms of concerns about standards and regulations in applications, nanochitosan is approved by the FDA (Food and Drug Administration) for biomedical and food applications. In addition, the quality control of nanochitosan and the cost of production are the main challenges limiting its wider adoption in agriculture. Nanochitosan lacks the ability to respond to environmental changes and stress, as it is susceptible to degradation by factors such as light, heat, and moisture. Advanced synthetic biology, advanced materials, and self-assembly nanotechnology to incorporate nanochitosan formulations will facilitate effective and precise sensing, response, and release capabilities, thereby improving the quality of agricultural products and minimizing the costs. Furthermore, based on a large amount of research, AI technology can explore a more comprehensive understanding of the growth-regulating effects, optimal costs, and stability of nanochitosan at different stages of plant growth [78].

## 7. Conclusions

Nanochitosan and its derivatives provide a sustainable alternative to traditional agrochemicals as a combination of biomass chitosan materials and nanotechnology. The reuse of nanochitosan as a waste product of the food industry has important implications for promoting zero waste of resources. This review discussed the sources, applications, mechanisms, and prospects of nanochitosan in agroecology and clarified the necessity for the advancement of agroecology. In this review, we not only focused on advantages in terms of sources, but also gave attention to the attractive potential of nanochitosan for the promotion of plant growth, pest/disease resistance, and drug delivery. We also summarized the mechanism of action and application scenarios of nanochitosan throughout the entire crop cycle, including its effects on crop yield, pesticide activity, and the reduction in agrochemical accumulation. Furthermore, the sources of nanochitosan and its effects on plant growth, antimicrobial properties, and delivery capacity were classified and discussed in detail. Finally, we discussed the challenges and provided an outlook for future nanochitosan research in agroecology, as well as the focus and trends.

## Figures and Tables

**Table 1:** Application of nanochitosan in promoting plant growth.

Site of Action	Morphology	Applied Crop	Effect	Function	References
Soil	Nanochitosan solution	Pinus edulis	Improves plant water and nutrient uptake	37% reduction in mortality	[21]
Soil	Nanochitosan particles	Corn	Increased bacterial diversity; increase in the number of OTUs (operational taxonomic units); increased enzyme activity (FDA (fluorescein diacetate), dehydrogenase, and alkaline phosphatase)	The composition, diversity, and richness of rhizosphere microbial communities were improved; improved soil health	[22]
Soil	Nanochitosan –Cu	Corn	Improved the activity of antioxidant enzymes in leaves, promoted the activity of maize source, and increased yield	Increased chlorophyll content (2-fold) and induced internode sucrose translocation (2.5–3.5-fold) to provide nutrients	[23]
Soil	Chitosan–urea nanoparticles	Potato	The fresh weight and dry weight of stems and leaves of potato were significantly increased; improved yield-related traits such as number of potatoes set and potato weight per plant	The contents of NH^4+^-N and NO^3−^-N in the soil were significantly reduced; the number of ammonia-oxidizing bacteria and nitrifying reducing bacteria in the soil decreased	[25]
Soil and foliar	Nanochitosan particles	Tomato	The density of root-knot nematodes decreased by 45.89–66.61% and the density of TMV (tobacco mosaic virus) decreased by 10.26–65.00%	Soil enzyme activity and increased plant defense enzyme activity	[9]
Soil and foliar	Nanochitosan solution	Tomatoes and potatoes	Control of bacterial wilt	Reduced morbidity and disease severity	[10]
Foliar	Nanochitosan–N fertilizer	Corn–soybean intercropping	The biological yield of maize increased, the occurrence of disease decreased by 78.93%, and the disease severity decreased by 71.85%; the biological yield of soybean increased, the occurrence of disease decreased by 81.64%, and the disease severity decreased by 77.63%	Promoted the absorption and utilization of nitrogen fertilizer and reduced nitrogen fertilizer loss	[5]
Foliar	Nanochitosan solution	Sesame	Improved resistance to Spodoptera litura, increased antioxidant enzyme activity, and improved nutritional quality	Activated the plant’s natural immunity to herbivorous insects and enhanced the production of defense metabolites	[6]
Foliar	Nanochitosan– gibberellin	Sorghum	Gibberellin-modified nanochitosan acted as a biostimulant to enhance the growth of sorghum under salt stress, and plant height, fresh weight and dry weight increased	Increased chlorophyll levels and increased antioxidant enzyme (POD (peroxidase) and SOD (superoxide dismutase)) activity	[6]
Foliar	Nanochitosan–K	Strawberry	The total yield increased, the market yield increased, and the fruit firmness increased significantly	Increased the content of soluble and exchangeable potassium in soils; increased total soluble solids, vitamin C levels, acidity, total sugars, and anthocyanin levels in the fruit	[7]
Foliar	Nanochitosan solution	Common beans	Plants treated with 62.5 mg/L nanochitosan showed higher chlorophyll content, plant height, fresh weight (stem and root), seed yield, and nutrient content	Increased organic matter, available nutrient content, and total bacterial count in soil and decreased Na% in fungal communities and plants	[8]
Foliar and seed	Nanochitosan–copper particles	Corn	Enhanced plant growth and yield, promoted nutrient uptake, enhanced plant defense response to disease, and improved plant growth and yield	Improved the activity of antioxidant enzymes and defense enzymes to control CLS (calcium lignosulfonate) disease	[20,28]
Seed	Nanochitosan–*Bacillus* spp.	Corn	Increased seed germination rate, plant height, root length, leaf area, fresh and dry weight, chlorophyll, carotenoids, and total sugar and protein content; promoted plant growth; enhanced defense response; and increased yield	Beneficial microbial communities in maize rhizosphere soil were improved	[11]
Seed	Nanochitosan solution	Cucumber	Increased germination rate (90%), enhanced seedling vigor (2665), and improved resistance to powdery mildew (66.6% disease protection)	Stimulated plant hormone content, induced the biosynthesis of defense-related enzymes, and enhanced plant growth	[12]
Seed	Nanochitosan–salicylic acid particles	Wheat	Increased the activity of seed reserve food remobilization enzyme; increased seedling vigor index (SVI) by 1.6 times, chlorophyll content by 1.46 times, and plant weight per pot	Promoted plant growth, improved antioxidant status, regulated reactive oxygen species (ROS) and malondialdehyde (MDA) content, and maintained cellular homeostasis	[13]
Seed	Nanochitosan	Corn	Improvement in growth parameters and soil quality, biomass increased and plant height (54%), leaf number (67.18%), photosynthetic pigment (65.62%), sugar (79.13%), protein (71.93%), phenol (136.57%), and flavonoids (167.61%)	The activity of antioxidant enzymes was increased: catalase (80.15%) and peroxidase (25.52%); the total number of soil bacteria increased (101%), phosphate (111%), the activity of soil enzymes was increased and dehydrogenase (94.88%), fluorescein diacetate (112%), and alkaline phosphatase (32.09%)	[29]
Seed	Nanochitosan– Ag	Wheat	Promoted seed germination, improved seed quality, and reduced the incidence of disease	The fungal load was reduced by 100%, the albumin content increased by 4.25 times, and the glutenin content increased by 5.78 times	[15]
Seed	Nanochitosan– salicylic acid solution	Lentil	Increased plant dry weight by 16% (after 35 days) and 40% (after 70 days)	Improved the mineral, soluble sugar, and pigment content, nitrogen content by about 40%, phosphorus content by about 52%; strengthened the defense mechanism (total phenols by about 2 times, peroxidase by about 7–69%, and polyphenol oxidase by about 16–50%)	[16]
Seed	Nanochitosan– polymer	Wheat	The aerial part increased by 9.8–15.3 mm; the underground part increased by 11.3–17.5 mm	Promoted seed germination and seedling growth and increased the germination rate by 3.3–4.0%	[17]
Seed	Nanochitosan solution	Corn	The germination rate increased to 96.97%, the plant height increased by 1.5 times, and the leaf area increased by 2 times	Promoted nutrient absorption, enhanced soil microbial activity, and improved the activity of soil health indicators such as dehydrogenase, fluorescein diacetate hydrolase, and alkaline phosphatase, and the activity was increased by 2 to 3 times	[18]
Seed	Nanochitosan– Pseudomonas particles	Tomato	Increases the growth percentage of tomato (46.62%) and reduces the percentage of disease (115.85%)	Soil enzyme activity was improved, including urease (411%), phosphatase (488%), catalase (765%), β-glucosidase (194%), and antifungal enzymes (chitinase 1666% and glucanase 89%); improved the activity of defense enzymes in tomato plants under infection, including superoxide dismutase (64%), polyphenol oxidase (80%), cell-wall-bound peroxidase (180%), and phenylalanine ammonia-lyase (180%)	[19]
